# Comparison of the VITEK REVEAL AST and Accelerate Pheno systems for fast antimicrobial susceptibility testing of gram-negative blood cultures at a large academic health system

**DOI:** 10.1128/jcm.01073-25

**Published:** 2025-12-17

**Authors:** Caroline Simmons-Williams, Luciano Soares, Deanna Becker, Amorce Lima, Laura Rowe, Dominic Uy, Heinz Salazar, Theresa Okeyo Owuor, Shivaramu Keelara, Richard Remington, Cecilia Carvalhaes, Suzane Silbert

**Affiliations:** 1 Tampa General Hospital 7829, Tampa, Florida, USA; 2 bioMerieux, St. Louis, Missouri, USA; Maine Medical Center Department of Medicine, Portland, Maine, USA

**Keywords:** bloodstream infections, fast antimicrobial susceptibility testing, VITEK REVEAL, Accelerate Pheno

## Abstract

Timely initiation of appropriate antimicrobial therapy is crucial for patients with Gram-negative (GN) blood stream infections. In this study, the performance of VITEK REVEAL (bioMérieux, USA), an FDA cleared *in vitro* diagnostic automated system for antimicrobial susceptibility testing (AST) directly from positive blood culture (BC), was compared to that of Accelerate Pheno (Accelerate Diagnostics, USA), as standard of care method. 128 GN positive BCs were analyzed according to manufacturer recommendations, comparing time to result (TTR) and AST results. The categorical agreement (CA) rate between VITEK REVEAL and Accelerate Pheno was 94.3% and the essential agreement (EA) rate was 96.0%. Very major discrepancies (VMD), major discrepancies (MD), and minor discrepancies (miD) rates between the systems were 7.5%, 0.5%, and 4.1%, respectively. Lastly, we observed a mean sequential TTR (reporting of AST results per antibiotic in real-time) on VITEK REVEAL of 6.1 h (3.0–8.2) and mean final TTR of 7.9 h (6.5–8.2), compared to Accelerate Pheno, with a significantly shorter mean final TTR of 7.1 h (6.8–7.7). Sequential TTR on VITEK REVEAL was significantly shorter for resistant isolates (those with ≥1 ‘Resistant’ antimicrobial-organism combination interpretation, using FDA STIC 2024 or CLSI M100 criteria) compared to susceptible/intermediate ones, with a mean difference of 1.4 h (*P*  <  0.001). Overall, compared to Accelerate Pheno, VITEK REVEAL displayed high %CA and %EA for AST of GN bacteria directly from positive BC. Also, unlike Accelerate Pheno, VITEK REVEAL reports MIC results in real time, allowing an earlier release of actionable information useful for antimicrobial stewardship.

**IMPORTANCE:**

Previous studies have compared the performance of the VITEK REVEAL, system for fast antimicrobial susceptibility testing (AST) to conventional, non-rapid microbiology methods. To evaluate how the VITEK REVEAL correlates to similarly available fast, direct-from-blood culture AST technologies, this study aimed to compare it to the Accelerate Pheno system, as standard of care method. High categorical agreement (94.3%) and essential agreement (96.0%) between the two systems were observed, underscoring their reliability. The VITEK REVEAL has the advantage of real-time AST reporting, unlike the Accelerate Pheno. Ultimately, this study supports the need for and continued optimization of fast diagnostic technologies for AST, contributing to the goal of advancing antimicrobial stewardship-focused treatment strategies and improving the prognosis of patients with bloodstream infections.

## INTRODUCTION

Complications from blood stream infections (BSI) caused by Gram-negative bacteria are one of the leading causes of hospital mortality in the United States (1, 2). If left untreated or improperly controlled, BSI can lead to sepsis, which is a dysregulated immunogenic host response to infection that can result in organ failure and death (2). Sepsis remains the leading cause of death in hospitals, with about 49 million cases and accounting for 19% of all deaths worldwide in 2017 (1). In high-income countries, the most common BSI pathogens include *Staphylococcus aureus*, *Escherichia coli*, *Klebsiella* spp., *Pseudomonas aeruginosa*, *Enterococci*, *Streptococci*, and coagulase negative *Staphylococci*, while in low-income countries, more than 50% of infections are caused by *Salmonella enterica* (3).

The management of BSI typically starts with broad-spectrum antimicrobials selected empirically upon diagnosis (4). Antimicrobial adjustment for effective treatment takes place after identification (ID) and antimicrobial susceptibility testing (AST) results for the causative isolate become available, which, depending on the methodology and species, can take up to or greater than 24–48 h (4). Additionally, unnecessary use of broad-spectrum antimicrobials or combination treatments can increase rates of adverse drug effects and contribute to the growing problem of antimicrobial resistant organisms (5, 6). Approximately half of patients with BSI are infected with antimicrobial resistant organisms, which often leads to treatment with improper empirical antimicrobial therapy (i.e., an antimicrobial for which the isolate from blood showed no *in vitro* susceptibility) and lower overall survival (7, 8). Thus, early confirmation of BSI and the consequent administration of appropriate antimicrobial therapy is critical to patient outcomes (9).

The current gold standard for the diagnosis of BSI consists of blood culture (BC) followed by species ID and AST (4, 10). Conventional methods for species ID rely on pure isolated colonies, which require a primary overnight culture of a positive BC on agar plates (4). Pure colonies are then subjected to Gram staining, followed by species ID by conventional automated systems (VITEK two and BD Phoenix) or matrix-assisted laser desorption ionization time of flight mass spectrometry (MALDI-TOF MS) (4). In tandem with species ID, AST is performed, utilizing methods and platforms such as broth microdilution, disk diffusion, ETEST gradient strips, and automated AST testing systems (VITEK 2, BD Phoenix, Affinity Biosensors LifeScale, Accelerate Pheno) (4, 11). Other methods for rapid species ID and detection of resistance markers also include BioFire BCID 2 Panel (bioMérieux), and droplet digital polymerase chain reaction panels for Gram-negative isolates (ddPCR-GNB) for species ID, and MALDI-AST for antimicrobial susceptibility testing (11–14).

The development of fast automated *in vitro* AST devices is a rapidly growing field. These new technologies test samples directly from positive blood culture and significantly reduce turnaround time for AST results (15). Direct from positive BC systems include Accelerate Pheno system (US FDA cleared and CE-IVD) (4, 16–22), Q-linea ASTar (CE-IVD) (4), AliFAX Alfred (CE-IVD) (4, 23), and bioMérieux VITEK REVEAL (US FDA cleared and CE-IVD) (5). Wide-scale development of these systems demonstrates the importance of timely and accurate diagnosis of Gram-negative BSI. Unfortunately, identification and antimicrobial susceptibility testing of causative pathogens proves challenging because of low circulating pathogen load (1–10 CFU/mL of blood), lengthy turnaround times for traditional microbiological bench culturing methods (greater than 24–48 h), fastidious organisms, and scope of pathogen coverage (5, 10, 13).

To address challenges in BSI diagnosis and AST, we evaluated the performance of the VITEK REVEAL (bioMérieux, US) system using the Accelerate Pheno (Accelerate Diagnostics, USA) as the reference standard of care method. The VITEK REVEAL system detects volatile metabolic byproducts (VMB) of microbial growth by exploiting a small molecule sensor technology using an array of sensors, which allow for real-time monitoring of AST microbial growth (10). In this study, we present the first clinical comparison between the VITEK REVEAL and Accelerate Pheno systems at a major healthcare facility, utilizing 158 clinical Gram-negative positive blood cultures to demonstrate agreement of AST results between both systems.

## MATERIALS AND METHODS

### Overview of clinical samples and processing

**Fig 1:**
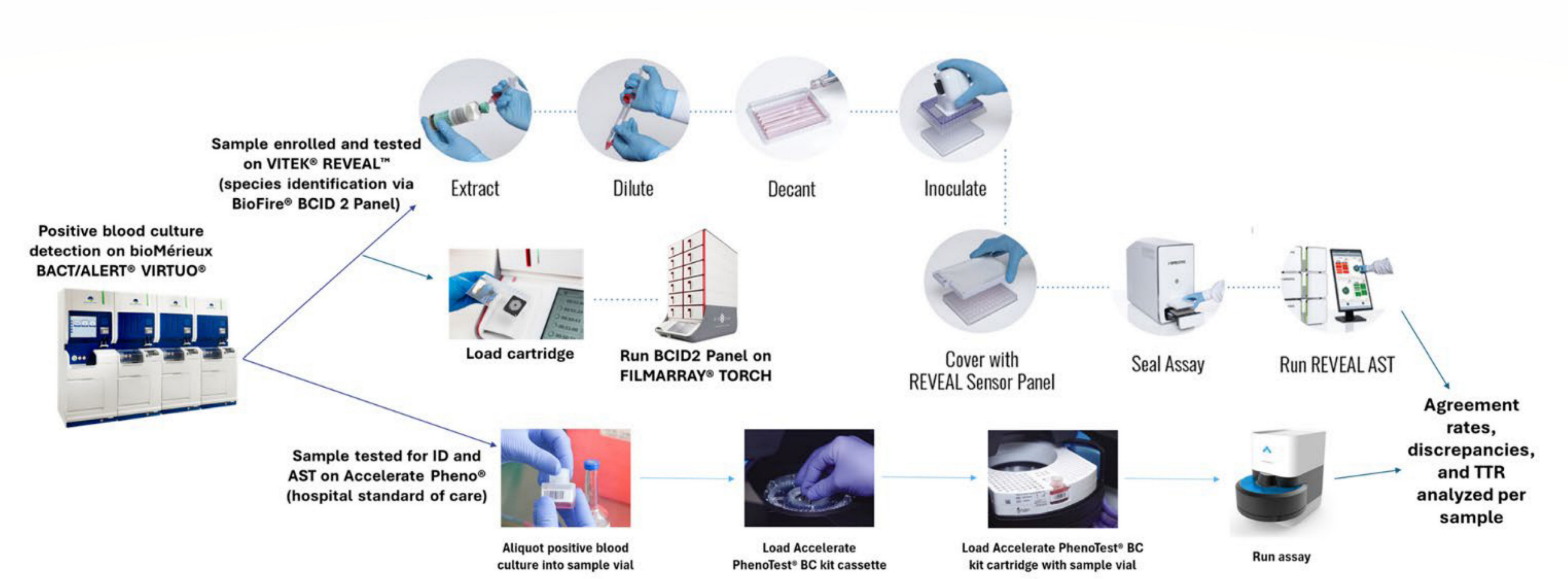
VITEK REVEAL vs Accelerate Pheno sample testing workflow. Testing workflow for enrolled samples on the Accelerate Pheno system, VITEK REVEAL AST, and BioFire BCID 2 Panel on the Torch System after positive identification on the BACT/ALERT VIRTUO. Agreement rates, discrepancies, and TTR were analyzed and compared between both systems.

Residual blood culture samples from patients with positive blood culture results at the Tampa General Hospital (Tampa, FL) from November 2023 to April 2024 were enrolled in this study. Samples were processed using the bioMérieux BACT/ALERT VIRTUO blood culture system with BACT/ALERT FA-PLUS, FN-PLUS, and/or PF-PLUS bottles (bioMérieux, Durham, NC). Prospective, de-identified residual positive blood cultures were selected for study inclusion. Following positivity on the BACT/ALERT VIRTUO, Gram staining was performed on each positive blood culture to confirm the presence of monomicrobial Gram-negative organisms. In addition, subculture plates were prepared to ensure sample purity. Samples were subsequently run on the Accelerate Pheno System for hospital standard of care/comparator testing within 30 min of positivity, and on the VITEK REVEAL AST within 16 h of sample positivity as outlined by the VITEK REVEAL AST Instructions For Use (IFU). VITEK REVEAL is optimized for use with positive blood culture samples within 16 h of sample positivity, in accordance with the protocol’s inclusion criteria and IFU. MIC calls on VITEK REVEAL were also deemed equivalent if tested anytime within the 16 h timeframe (stability has not been determined outside of the 16 h timeframe). Differences in run time between the Accelerate Pheno and VITEK REVEAL would not affect test results. An overview of the VITEK REVEAL and Accelerate Pheno testing workflows is included in Fig. 1. Samples included in the study were positive to one of the following bacterial species: *Klebsiella oxytoca*, *Klebsiella pneumoniae*, *Klebsiella aerogenes*, *Escherichia coli*, *Enterobacter cloacae* complex, *Citrobacter freundii*, *Citrobacter koseri*, *Proteus mirabilis*, *Proteus vulgaris*, *Serratia marcescens*, *Pseudomonas aeruginosa*, or *Acinetobacter baumannii* complex/group. All tested blood culture samples were frozen in two aliquots (in TSB [tryptic soy broth] with 15% glycerol, maintained at ≤ −70°C) for error resolution testing via reference broth microdilution (BMD), when needed. The study excluded samples with no species ID, IDs that were not included on the VITEK REVEAL AST (IFU), polymicrobial samples, no MIC values for most/all drugs due to technical errors, positive blood cultures > 16 h post-positivity, and non-Gram-negative bacilli, i.e., yeast and Gram-positive bacteria (Fig. S1).

### Accelerate Pheno, BioFire BCID 2, and VITEK REVEAL AST Testing

Accelerate Pheno (Software Version 1.5.0.30) testing was performed with the Accelerate PhenoTest BC Kit (US-IVD) according to manufacturer protocols. A volume of 0.5 mL of direct positive blood culture sample was loaded within 30 min of culture positivity into the Accelerate PhenoTest BC kit sample vial and loaded onto the Accelerate Pheno reagent cartridge before the run started in accordance with hospital standard of care protocol. Each culture tested was also inoculated on solid media to assess purity, and plate growth was analyzed for consistency with ID and AST on Accelerate Pheno.

VITEK REVEAL testing was performed using the GN BC-AST RUO (research use only) panel on the VITEK REVEAL AST System. A 1:1,000 dilution was prepared by directly adding 25 µL of positive blood culture into a 25 mL vial of Pluronic water (provided by bioMérieux). The vial was inverted 10 times to mix, and the suspension was decanted into a RENOK tray (bioMérieux). A RENOK rehydrator (Beckman Coulter) was used to transfer 115 µL of the sample into each well of the VITEK REVEAL GN BC-AST panel. The AST panel was sealed with the VITEK REVEAL Sensor panel and loaded onto the VITEK REVEAL for testing and analysis. Following inoculation of the GN BC-AST panel, a purity plate was prepared with the remaining Pluronic water/positive blood culture dilution to ensure a pure culture was used for testing. When technical errors with the VITEK REVEAL system were observed during testing, the sample was retested (if within the acceptable blood culture post-positivity range of <16 h). The BioFire BCID 2 Panel on the Torch System was used according to manufacturer recommendation, in tandem with the VITEK REVEAL AST System, for rapid species identification.

### Quality control

Testing on the VITEK REVEAL was conducted with adequate quality control (QC) per manufacturer’s Instructions for Use (IFU). Out-of-range QC results were retested and, if repeated testing results were still out of range, the study paused for troubleshooting.

### Discrepancy testing by reference broth microdilution

Discrepant isolates (corresponding to very major [VMD], major [MD], and minor [miD] discrepancies) on the VITEK REVEAL vs the Accelerate Pheno were submitted for reference broth microdilution at a reference laboratory (JMI-Element, USA) for discrepancy analysis following CLSI M100 ED34 and CLSI M07 documents (24, 25). All discrepant samples were tested in triplicate and appropriate ATCC quality control strains were tested concurrently with each drug per day of testing.

### Data capture and analysis

De-identified device data (ID, AST, time to result [TTR], and other study data) for both devices were entered into a cloud-based electronic data capture (EDC) system. The analysis began by excluding samples that did not meet the eligibility criteria. Minimum inhibitory concentration (MIC) results generated by the VITEKREVEAL were compared to those from the Accelerate Pheno for each antimicrobial-microorganism combination tested. Combinations lacking MIC values or affected by claims, reporting suppression, or intrinsic resistance were excluded. Combinations with reported MICs were further evaluated for claims, limitations, and interpretation to Susceptible (S), Intermediate (I), or Resistant (R). Limitations were defined according to the VITEK REVEAL and the Accelerate Pheno IFUs, which recommend alternative testing methods when necessary for patient care. Only antimicrobial-microorganism combinations that met all criteria—MIC reported, no limitations, interpretable MICs, and claimed on both instruments—were included in the final analysis (Fig. S1). To ensure consistency and avoid inappropriate comparisons, the same interpretive breakpoints were applied across all systems. MIC results from both instrument reports and BMD were interpreted using standardized criteria from the FDA STIC 2024 published on 20 June 2024 (26). For antimicrobial agents without FDA-designated breakpoints, interpretive criteria from CLSI M100, 34th Edition (24), were applied when recognized by the FDA (Table S1). Essential agreement (EA), categorical agreement (CA), and discrepancy (VMD, MD, and miD) rates were evaluated. For each discrepant isolate, the original VITEKREVEAL AST and the Accelerate Pheno results were compared with the BMD mode result.

MIC calls and categorical interpretations were visible on the VITEK REVEAL user interface in real-time after organism identification was entered into the system. Accelerate Pheno results were available after completion of the assay. The final TTR (measured from the time a sample was loaded onto the instrument until the final antimicrobial result was displayed upon completion of the assay) was compared between the VITEKREVEAL AST and the Accelerate Pheno systems. To evaluate differences in sequential TTR by susceptibility category, isolates were classified based solely on VITEK REVEAL results interpreted as described above. The bacterial isolates were split into S/I and R categories based on each antimicrobial-organism combination criteria and the TTR was analyzed by antimicrobial. Differences in proportions were tested with two-sample *z*-test. Median differences in TTR were tested with Wilcoxon rank-sum test for unpaired comparisons and the Wilcoxon signed-rank test for paired samples. Mean differences in TTR were tested with *t*-test for unpaired comparisons and paired *t*-test for paired comparisons. All data analysis was performed with the R statistical programming language (version 4.4.1) (27).

## RESULTS

### Overview of sample species and antimicrobials

Out of 158 de-identified, positive Gram-negative blood culture samples included in the study (Table S2), 30 were excluded from analysis (Fig. S1). The remaining 128 analyzed samples consisted of 115 (89.8%) Enterobacterales species and 13 (10.2%) *P*. *aeruginosa* (Table S2), identified by BioFire BCID 2 Panel on the Torch System. Two *Citrobacter* spp. isolates unable to be identified via BCID 2 Panel were instead identified by MALDI-ToF.

Antimicrobial results selected for performance analysis were prioritized based on their availability on both the VITEK REVEAL and Accelerate Pheno AST panels. Six antimicrobials available on the VITEK REVEAL GN BC AST RUO panel were not available on the Accelerate Pheno AST panel and were excluded from evaluation. The remaining 14 antimicrobials available on both AST systems were included in the final analysis (Table S1).

### Performance of VITEK REVEAL vs Accelerate Pheno systems

Overall, the essential agreement (EA) and categorical agreement (CA) between both systems were 96.0% (1,353/1,410) and 94.3% (1,329/1,410), respectively (Table 1). The EA and CA were 96.1% and 94.4%, respectively, across all Enterobacterales species, and 94.2% and 91.9% for *Pseudomonas aeruginosa*. For the Enterobacterales group, only ampicillin/sulbactam (EA, 63.8%; CA 53.6%) fell below the 90% threshold. For *Pseudomonas aeruginosa*, aztreonam and cefepime had EA and CA below 90%, while meropenem had a CA below 90%. Species-level breakdown for Enterobacterales is provided in Table S3. The following species showed overall EA and/or CA rates below 90%: *C. koseri* (EA, 83.3%), *K. oxytoca* (EA, 87.5%; CA, 87.5%).

**TABLE 1:** Performance of the VITEK REVEAL vs Accelerate Pheno systems by antimicrobial for Enterobacterales group and *P. aeruginosa^b^^,c^*

	No. of strains with Accelerate Pheno as reference			Agreement		Discrepancies		
	S	I	R	% EA (*n*/Total)	% CA (*n*/Total)	% VMD (*n*/*R*)	% MD (*n*/S)	miD (*n*/Total)
*Enterobacterales (n = 115)*								
*Amikacin*	109	0	0	99.1 (108/109)	100.0 (109/109)	NA^*d*^	0.0 (0/109)	0.0 (0/109)
*Ampicillin/Sulbactam*	28	5	36	63.8 (44/69)	53.6 (37/69)	22.2 (8/36)	0.0 (0/28)	34.8 (24/69)
*Aztreonam*	65	1	28	95.8 (91/95)	93.7 (89/95)	7.1 (2/28)	3.1 (2/65)	2.1 (2/95)
*Cefepime^a^*	75	3	21	97.0 (96/99)	92.9 (92/99)	0.0 (0/21)	0.0 (0/75)	7.1 (7/99)
*Ceftazidime*	65	7	26	96.9 (95/98)	89.8 (88/98)	11.5 (3/26)	0.0 (0/65)	7.1 (7/98)
*Ceftazidime/Avibactam*	106	0	0	100.0 (106/106)	100.0 (106/106)	NA	0.0 (0/106)	0.0 (0/106)
*Ceftriaxone*	72	0	33	99.0 (104/105)	99.0 (104/105)	0.0 (0/33)	1.4 (1/72)	0.0 (0/105)
*Ciprofloxacin*	65	3	33	96.0 (97/101)	90.1 (91/101)	0.0 (0/33)	3.1 (2/65)	7.9 (8/101)
*Ertapenem*	91	0	2	100.0 (92/92)	98.9 (91/92)	0.0 (0/1)	0.0 (0/91)	1.1 (1/92)
*Gentamicin*	85	2	19	98.1 (103/105)	97.1 (102/105)	10.5 (2/19)	0.0 (0/85)	1.0 (1/105)
*Meropenem*	101	0	0	98.0 (99/101)	100.0 (101/101)	NA	0.0 (0/101)	0.0 (0/101)
*Piperacillin/* *Tazobactam^a^*	71	0	4	93.3 (70/75)	97.3 (73/75)	25.0 (1/4)	0.0 (0/71)	1.3 (1/75)
*Tobramycin*	95	3	13	99.1 (110/111)	98.2 (109/111)	0.0 (0/13)	0.0 (0/95)	1.8 (2/111)
*Trimethoprim/* *Sulfamethoxazole*	49	0	9	98.3 (57/58)	100.0 (58/58)	0.0 (0/9)	0.0 (0/49)	0.0 (0/58)
*Total Antimicrobials*	1,077	24	223	96.1 (1,272/1,324)	94.4 (1,250/1,324)	7.2 (16/223)	0.5 (5/1,077)	4.0 (53/1,324)
*Pseudomonas aeruginosa (n = 13)*								
*Amikacin*	13	0	0	100.0 (13/13)	100.0 (13/13)	NA	0.0 (0/13)	0.0 (0/13)
*Aztreonam*	8	1	3	84.6 (11/13)	76.9 (10/13)	33.3 (1/3)	0.0 (0/8)	15.4 (2/13)
*Cefepime*	8	0	0	75.0 (6/8)	87.5 (7/8)	NA	12.5 (1/8)	0.0 (0/8)
*Ciprofloxacin*	12	0	2	100.0 (13/13)	100.0 (13/13)	0.0 (0/1)	0.0 (0/12)	0.0 (0/13)
*Gentamicin*	12	2	0	92.3 (12/13)	92.3 (12/13)	NA	0.0 (0/12)	7.7 (1/13)
*Meropenem*	11	2	2	100.0 (13/13)	84.6 (11/13)	0.0 (0/1)	0.0 (0/11)	15.4 (2/13)
*Tobramycin*	13	0	0	100.0 (13/13)	100.0 (13/13)	NA	0.0 (0/13)	0.0 (0/13)
*Total Antimicrobials*	77	4	5	94.2 (81/86)	91.9 (79/86)	20.0 (1/5)	1.3 (1/77)	5.8 (5/86)
*All organisms (n = 128)*	1,154	28	228	96.0 (1,353/1,410)	94.3 (1,329/1,410)	7.5 (17/228)	0.5 (6/1,154)	4.1 (58/1,410)

^*a*^ Susceptible dose-dependent (SDD) breakpoints apply to Enterobacterales when tested against cefepime and piperacillin-tazobactam.

^*b*^ Results shown are prior to discrepancy resolution.

^*c*^ The number of reported strains varies by antimicrobial, as combinations without MIC values or affected by claims, suppression, or intrinsic resistance were excluded. Susceptible (S), intermediate (I), or resistant (R). % EA (*n*/Total) (percent essential agreement); % CA (*n*/Total) (percent categorical agreement); VMD (percent very major discrepancy); MD (percent major discrepancy); miD (percent minor discrepancy); – symbol = not applicable.

^*d*^ NA, not available.

### Overview of discrepancy rates

Across all organisms, the rates of VMD, MD, and miD were 7.5% (*n* = 17/228), 0.5% (*n* = 6/1,154), and 4.1% (*n* = 58/1,410), respectively. The performance by antimicrobial and bacterial species is displayed in Table 1 and Table S3. VMDs for the Enterobacterales group were observed for ampicillin/sulbactam (*n* = 8), aztreonam (*n* = 2), ceftazidime (*n* = 2), gentamicin (*n* = 2), and piperacillin/tazobactam (*n* = 1), while MDs were detected for aztreonam (*n* = 2), cefepime (*n* = 1), ceftriaxone (*n* = 1), and ciprofloxacin (*n* = 2). Most of the minor discrepancies were observed with ampicillin/sulbactam and are described in Table S4. When stratified by species, *E. coli* (*n* = 14), *K. oxytoca* (*n* = 1), *P. mirabilis* (*n* = 1), and *P. aeruginosa* (*n* = 1) accounted for all VMDs (Table 1; Tables S3 and S4). Most of the MDs were from *E. coli* (*n* = 4) and for the following antimicrobials: aztreonam (*n* = 2), ceftriaxone (*n* = 1), and ciprofloxacin (*n* = 1). Additionally, *P. aeruginosa* had one MD for aztreonam. Of the 58 miD cases, 39 included *E. coli* (which accounted for the majority of the miDs [39/58]), two included *K. aerogenes*, six included *K. pneumoniae*, five included *P. aeruginosa,* five included *P. mirabilis*, and one included *E. cloacae* complex (Table S4).

### Discrepancy resolution by reference broth microdilution

When compared to reference BMD results, 54.3% of all discrepancies were resolved in favor of the VITEK REVEAL compared to 33.3% for Accelerate Pheno (*P*. value = 0.05681) (Table 2). VMDs were resolved in favor of the VITEK REVEAL in 47.1% (*n* = 8), compared to only 11.8% (*n* = 2) in favor of the Accelerate Pheno (*P*. value = 0.1094). The remaining 7 VMDs (41.7%) were unable to be resolved for either system. On the other hand, 4 out of 6 (66.7%) MDs were resolved in favor of the Accelerate Pheno while one MD was resolved in favor of VITEK REVEAL (p. value = 0.375). The remaining MD was unresolved for either platform. A total of 60.3% (*n* = 35) of the miDs were resolved in favor of the VITEK REVEAL compared to 36.2% (*n* = 21) for Accelerate Pheno (*P*. value = 0.0814) (Table 2). Further assessment of the miDs revealed that VITEK REVEAL more often undercalled resistance, reporting Intermediate in 14 cases where BMD indicated Resistant. In contrast, Accelerate Pheno more frequently overcalled resistance, reporting Resistant in 11 cases where BMD indicated Susceptible (1 case) or Intermediate (10 cases). Additionally, Accelerate Pheno undercalled resistance in three cases, while VITEK REVEAL overcalled resistance in two cases (Table S4).

**TABLE 2:** Discrepancies resolved by BMD by antimicrobial^*a*^

Antimicrobial	All discrepancies (*n*)				Very major discrepancies (*n*)				Major discrepancies (*n*)				Minor discrepancies (*n*)			
	VR vs AP	Resolved by BMD		Not resolved by BMD	VR vs AP	Resolved by BMD		Not resolved by BMD	VR vs AP	Resolved by BMD		Not resolved by BMD	VR vs AP	Resolved by BMD		Not resolved by BMD
		VR	AP			VR	AP			VR	AP			VR	AP	
Ampicillin/ Sulbactam	32	15	13	4	8	3	2	3	0	0	0	0	24	12	11	1
Aztreonam	9	4	3	2	3	2	0	1	2	0	1	1	4	2	2	0
Cefepime	8	6	1	1	0	0	0	0	1	1	0	0	7	5	1	1
Ceftazidime	10	7	2	1	3	2	0	1	0	0	0	0	7	5	2	0
Ceftriaxone	1	0	1	0	0	0	0	0	0	0	0	0	0	0	0	0
Ciprofloxacin	10	5	5	0	0	0	0	0	1	0	1	0	8	5	3	0
Ertapenem	1	0	1	0	0	0	0	0	0	0	0	0	1	0	1	0
Gentamicin	4	2	1	1	2	1	0	1	1	0	1	0	2	1	1	0
Meropenem	2	2	0	0	0	0	0	0	0	0	0	0	2	2	0	0
Piperacillin/ Tazobactam	2	1	0	1	1	0	0	1	1	0	1	0	1	1	0	0
Tobramycin	2	2	0	0	0	0	0	0	0	0	0	0	2	2	0	0
Overall (*n*)	81	44	27	10	17	8	2	7	6	1	4	1	58	35	21	2
Overall (%)	NA^*b*^	54.3	33.3	12.3	NA	47.1	11.8	41.2	NA	16.7	66.7	16.7	NA	60.3	36.2	3.4

^*a*^ *n,* number of isolates; VR, VITEK REVEAL; AP, Accelerate Pheno.

^*b*^ NA, not applicable.

### Final and sequential time to result on VITEK REVEAL AST and Accelerate Pheno systems

VITEK REVEAL reports TTR both sequentially by drug in real-time following input of sample species ID and as a final TTR. The Accelerate Pheno system does not allow for sequential, real-time reporting of AST results; therefore, no comparison was made with the VITEK REVEAL for this parameter.

The median, mean, and range of the final TTR for both systems by species group are summarized in Table 3. The mean final TTR for all antimicrobial-organism combinations on the VITEK REVEAL was 7.9 h (range 6.5–8.2 h, median 8.0 h), compared to a mean final TTR of 7.1 h (range 6.8–7.7 h, median 7.1 h–) on the Accelerate Pheno. The difference in mean final TTR between both systems was statistically significant (*P* < 0.001). Mean final TTR varied by species on the VITEK REVEAL, with *K. pneumoniae* and *P. aeruginosa* both having a final TTR of 6.5 h, *E. coli* a final TTR of 8.0 h, and the “Other Enterobacterales” group (consisting of *C. koseri*, *C. freundii*, *E. cloacae* complex, *K. aerogenes*, *K. oxytoca*, *P. mirabilis*, and *S. marcescens*) having a mean final TTR of 7.1 h. In contrast, the Accelerate Pheno mean final TTR was consistent across Enterobacterales species at 7.1 h each. *P. aeruginosa* had a mean final TTR of 7.0 h on the Accelerate Pheno.

**TABLE 3:** Final vs sequential time to result (TTR) on the VITEK REVEAL and final TTR for Accelerate Pheno, by species groups for all antimicrobials

Species	Number	VITEK REVEAL			VITEK REVEAL			Accelerate Pheno			VITEK REVEAL vs Accelerate Pheno (difference)			
		Sequential reporting TTR^*a*^			Final TTR			Final TTR			Final TTR			
		Median	Mean	Range	Median	Mean	Range	Median	Mean	Range	Median	*P* value	Mean	*P* value
*E. coli*	60	6.2	6.0	3.1–8.2	8.0	8.0	8.0–8.2	7.1	7.1	7.02–7.08	1.0	1.42E−11	1.0	2.89E−74
*K. pneumoniae*	24	6.5	6.1	3.0–6.6	6.5	6.5	6.5–6.6	7.1	7.1	7.02–7.12	−0.6	1.80E−05	−0.5	2.23E−23
Other Enterobacterales^*b*^	31	6.5	6.6	3.0–8.2	8.0	7.5	6.5–8.2	7.1	7.1	6.80–7.68	0.9	8.17E−04	0.4	2.29E−03
*P. aeruginosa*	13	5.8	5.7	4.0–6.6	6.5	6.5	6.5–6.6	7.0	7.0	6.97–7.03	−0.5	1.60E−03	−0.5	3.29E−12
All organisms	128	6.5	6.1	3.0–8.2	8.0	7.5	6.5–8.2	7.1	7.1	6.8–7.7	0.9	4.79E−12	0.4	2.23E−09

^*a*^ The Accelerate Pheno system does not allow for sequential reporting of AST results; therefore, no comparison was made with the VITEK REVEAL for this parameter.

^*b*^ Other Enterobacterales group includes *C. koseri, C. freundii, E. cloacae* complex, *K. aerogenes, K. oxytoca, P. mirabilis*, and *S. marcescens*. TTR, time to result.

The VITEK REVEAL, mean TTR for sequential reporting of real-time MIC results was 6.1 h (range 3.0–8.2, median 6.5 h) (Table 3). The TTR for sequential reporting on the VITEK REVEAL was further stratified by resistant (*n* = 197) and susceptible/intermediate (*n* = 1,213) antimicrobial-organism combinations (Table 4). The resistant group had a mean sequential TTR of 4.9 h (range 3.0–8.1 h, median 4.7 h) compared to the susceptible/intermediate group with a mean sequential TTR of 6.3 h (4.0–8.2 h, median 6.5 h). Seven antimicrobials (aztreonam, cefepime, ceftazidime, ceftriaxone, ciprofloxacin, gentamicin, and tobramycin) showed a statistically significant difference (*P* < 0.05) for both the mean and median sequential TTRs in the resistant vs susceptible/intermediate groups. Overall, a 1.4 h difference in mean sequential TTR was observed between resistant and susceptible/intermediate groups (*P* < 0.001).

**TABLE 4:** Breakdown of time to result by antimicrobials for resistant, and susceptible and intermediate strains on the VITEK REVEAL^*a*^

Antimicrobials	All data				Resistant				Susceptible/Intermediate				Resistant vs susceptible/intermediate (difference)			
	*n*	Median	Mean	Range	*n*	Median	Mean	Range	N	Median	Mean	Range	Median	*P* value	Mean	*P* value
Amikacin	122	6.5	6.1	4.0–8.2	0	NA^*b*^	NA	NA	122	6.5	6.1	4.0–8.2	NA	NA	NA	NA
Ampicillin/Sulbactam	69	6.5	6.4	3.2–6.6	11	6.5	5.8	3.2–6.6	58	6.5	6.5	6.0–6.6	0.0	2.45E−01	−0.7	1.11E−01
Aztreonam	108	6.1	6.0	3.0–8.0	30	6.0	5.4	3.0–6.6	78	6.3	6.3	6.0–8.0	−0.3	7.72E−05	−0.9	4.29E−04
Cefepime	107	6.5	6.3	3.3–8.0	22	6.0	5.8	3.3–6.6	85	6.5	6.5	6.0–8.0	−0.5	3.22E−04	−0.7	5.01E−03
Ceftazidime	98	5.5	5.6	3.0–6.6	24	5.5	5.1	3.0–6.6	74	5.6	5.8	5.5–6.6	−0.1	2.54E−02	−0.7	1.22E−02
Ceftazidime/Avibactam	106	6.5	6.4	6.0–6.6	0	NA	NA	NA	106	6.5	6.4	6.0–6.6	NA	NA	NA	NA
Ceftriaxone	105	6.0	5.6	3.0–8.1	34	3.6	3.9	3.0–8.1	71	6.2	6.5	6.0–8.1	−2.5	7.03E−15	−2.5	4.77E−17
Ciprofloxacin	114	5.5	5.4	3.0–8.2	36	4.5	4.6	3.0–8.0	78	5.5	5.7	4.0–8.2	−1.0	1.42E−07	−1.1	1.06E−05
Ertapenem	92	6.5	6.7	6.5–8.1	0	NA	NA	NA	92	6.5	6.7	6.5–8.1	NA	NA	NA	NA
Gentamicin	118	4.5	5.2	4.0–8.2	16	4.3	4.3	4.1–4.7	102	4.8	5.4	4.0–8.2	−0.5	4.01E−02	−1.0	3.41E−12
Meropenem	114	8.0	7.5	6.5–8.2	0	NA	NA	NA	114	8.0	7.5	6.5–8.2	NA	NA	NA	NA
Piperacillin/Tazobactam	75	8.0	7.7	6.5–8.2	2	8.0	8.0	8.0–8.0	73	8.0	7.7	6.5–8.2	0.0	9.56E−01	0.3	5.93E−05
Tobramycin	124	5.0	5.3	3.3–8.2	13	4.8	4.6	3.3–5.1	111	5.0	5.4	4.8–8.2	−0.2	1.07E−04	−0.8	3.00E−04
Trimethoprim/Sulfamethoxazole	58	4.3	4.9	3.2–6.6	9	4.8	4.9	3.2–6.5	49	4.3	4.9	4.0–6.6	0.5	9.31E−01	0.0	9.40E−01
Overall	NA	6.5	6.1	3.0–8.2	NA	4.7	4.9	3.0–8.1	NA	6.5	6.3	4.0–8.2	−1.8	1.40E−40	−1.4	8.17E−34

^*a*^ *n,* number of isolates tested per drug.

^*b*^ NA, not applicable.

## DISCUSSION

Blood culture standard of care testing at Tampa General Hospital is performed on the BACT/ALERT VIRTUO (bioMérieux), followed by Gram staining and subsequent species identification and AST on the Accelerate Pheno System. Although the performance of the newly FDA-cleared VITEK REVEAL has been compared to multiple conventional AST systems, data are lacking on its performance compared to other fast AST systems that obtain results directly from BC (11, 28, 29). Additionally, with the recent bankruptcy of Accelerate Diagnostics, a direct comparison between VITEK REVEAL and Accelerate Pheno is particularly needed for facilities that, in the near future, may need to switch from Accelerate Pheno to other fast AST platforms. Therefore, data comparing both of these systems will allow for more informed decision making on how modifying AST systems may affect workflow, turnaround times, AST results, and clinical utilization. In this study, we provide the first clinical evaluation and head-to-head comparison of the VITEK REVEAL and the Accelerate Pheno systems.

A total of 1,410 antimicrobial-organism combinations were analyzed, with an overall EA and CA rates between VITEK REVEAL vs Accelerate Pheno systems of 96.0% and 94.3%, respectively. Importantly, only antimicrobials reported for both systems were included in this analysis. A total of six antimicrobials reported by VITEK REVEAL could not be evaluated as they were not claimed on the Accelerate Pheno at the time of the study. Notably, all antimicrobials showed either EA or CA rates > 90%, except for ampicillin/sulbactam, which had an EA of 63.8% and CA of 53.6%. Tibbetts et al. also encountered low CA between the VITEK REVEAL and Sensititre (ThermoFisher Scientific) for ampicillin/sulbactam (66.3%), albeit the EA was 98.8% (11). They speculated that this could be attributed to a high number of strains with MIC values bordering ampicillin/sulbactam breakpoints, demonstrated by a high number of strains in the intermediate susceptibility category (11). Our data also showed a high number of samples in the intermediate susceptibility category (*n* = 19; 46.3%) for ampicillin/sulbactam on the VITEK REVEAL. Additionally, Ostermann et al. reported a CA of 85.9% for amoxicillin/clavulanic acid between the VITEK REVEAL and DxM MicroScan WalkAway system and suggested this may be due to testing combinations of a beta-lactams plus a beta-lactamase inhibitor in strains with an MIC near breakpoints (29). Future studies are needed to further investigate the reasoning behind this observation.

VMD, MD, and miD rates were 7.5% (*n* = 17/228), 0.5% (*n* = 6/1154), and 4.1% (*n* = 58/1410) between VITEK REVEAL vs Accelerate Pheno systems, respectively, before discrepancy resolution. Ampicillin/sulbactam accounted for eight of the 17 VMDs, potentially due to reasons previously discussed above. *E. coli* accounted for the majority of the VMDs (14/17; 82.4%), MDs (4/6; 66.7%), and miDs (39/58; 67.2%). This may be due to the significantly higher number of *E. coli* samples enrolled in the study as opposed to other bacterial species, accounting for almost half samples (60/128; 46.9%) analyzed. In instances of discordance between the two instruments, BMD results more often aligned with the VITEK REVEAL, favoring it in over half of the cases.

Minor discrepancies were more often associated with undercalling resistance (14 cases on the VITEK REVEAL and 3 cases on the Accelerate Pheno)—reporting “Intermediate” when reference BMD indicated “Resistant.” While typically less critical than major discrepancies or very major discrepancies, this may delay appropriate therapy. This underscores the importance of reviewing fast AST results with the support of antimicrobial stewardship teams to help ensure timely interpretation and intervention, especially when intermediate results are reported.

The primary advantages of fast AST systems to aid patient treatment plans and care are their ability to provide antimicrobial susceptibility profiles for isolates in shorter timeframes compared to traditional microbiology culturing methods. The mean final TTR for all drugs on the VITEK REVEAL was 7.9 h, while on the Accelerate Pheno was 7.1 h. As opposed to Accelerate Pheno, AST results are available on the VITEK REVEAL in real-time via sequential TTR, allowing for an earlier release of actionable information useful for antimicrobial stewardship and treatment strategies. The mean sequential TTR for all species on VITEK REVEAL was 5.9 h, with *P. aeruginosa* and *E. coli* strains having the lowest mean sequential TTR of 5.6 h and 5.8 h, respectively. Further investigation for species-specific sequential TTR on the VITEK REVEAL is needed to determine this significance. Overall, sequential reporting of MIC results on VITEK REVEAL offers a significant advantage versus Accelerate Pheno, most notably in high volume facilities where high resistance prevalences complicate accurate treatment regimens and encourage frequent reevaluations in a timely manner (10). We plan to further investigate the clinical implications of sequential reporting and its potential utility in antimicrobial decision-making in future studies.

When analyzing sequential TTR by resistance groups (resistant and susceptible/intermediate organisms), our data corroborate findings by Menchinelli et al. (30), showing that results on resistant isolates are available 1.2 h sooner than results on susceptible/intermediate organisms. Notably, ceftriaxone TTR difference between resistant vs susceptible/intermediate groups was 2.5 h. A mean TTR for ceftriaxone resistance of <4 h is an important finding for management of infections caused by extended spectrum β-lactamases (ESBL)-producing organisms.

Delays in the introduction of appropriate antimicrobial treatments for BSI are associated with increased 30-day mortality after 12 h from blood culture collection, therefore, providing rapid microbiological diagnostics from blood cultures of resistant strains is imperative (28). This current study was limited by its smaller sample size (*n* = 128), represented by many *E. coli* isolates in the data set. Future studies are needed with larger sample sizes to alleviate this species bias and allow for more diversity. Additionally, while this study compared TTR and agreement between VITEK REVEAL and Accelerate Pheno, both hands-on time/ease of use by laboratory professionals and costs of implementation were not studied and require further investigation. Furthermore, neither system was clinically validated for CLSI breakpoints prior to applying them when FDA breakpoints were not available. A comparison of the scalability and variety of drug menus offered on the VITEK REVEAL vs Accelerate Pheno is needed to determine how both systems compare in improving clinical performance and utility at the hospital.

In conclusion, fast tests for AST directly from blood culture have made substantial strides towards improving the assessment and treatment of patients with Gram negative bloodstream infections. VITEK REVEAL has recently obtained US FDA 510(k) clearance (31) and, combined with its high categorical agreement with Accelerate Pheno and real-time AST reporting, is a reliable platform for managing patient treatment strategies and antimicrobial stewardship in healthcare facilities.

## Data Availability

Data available as supplemental material.

## SUPPLEMENTAL MATERIAL

Supplemental material jcm.01073-25-s0001.docx Figure S1; Tables S1 to S4.

ASM does not own the copyrights to Supplemental Material that may be linked to, or accessed through, an article. The authors have granted ASM a non-exclusive, world-wide license to publish the Supplemental Material files. Please contact the corresponding author directly for reuse.
