# Supplementary material for: Comparison of the VITEK REVEAL AST and Accelerate Pheno systems for fast antimicrobial susceptibility testing of gram-negative blood cultures at a large academic health system
Source: J Clin Microbiol. 2025 Dec 17;64(1):e01073-25. doi: 10.1128/jcm.01073-25 (PMC12802176; doi:10.1128/jcm.01073-25)
Supplement: Supplemental material — Figure S1; Tables S1 to S4. [file jcm.01073-25-s0001.docx]

**Figure S1** Decision tree outlining the inclusion and exclusion criteria for clinical samples and antimicrobial–microorganism combinations used in performance analysis.

^1^Limitations were defined according to the VITEK® REVEAL™ and the Accelerate Pheno® IFUs, which recommend alternative testing methods when necessary for patient care.

VR - VITEK® REVEAL™, AP - Accelerate Pheno®; EA- essential agreement); CA - categorical agreement; TTR – time to result; MIC minimum inhibitory concentration

**TABLE S1** Antimicrobials tested on the VITEK® REVEAL™ vs. Accelerate Pheno®

|  |  |  | **Enterobacterales FDA BPs^1^** | | | ***Pseudomonas aeruginosa* FDA BPs^1^** | | |
| --- | --- | --- | --- | --- | --- | --- | --- | --- |
| **Antimicrobial** | **VITEK® REVEAL™^2^** | **Accelerate Pheno®** | **S** | **I** | **R** | **S** | **I** | **≥R** |
| Amikacin^5^ | x | x | ≤16 | 32 | ≥64 | ≤16 | 32 | ≥64 |
| Amoxicillin/Clavulanate | x | NA | NA | NA | NA | NA | NA | NA |
| Ampicillin/Sulbactam^3^ | x | x | ≤8/4 | 16/8 | ≥32/16 | - | - | - |
| Aztreonam^3,5,6^ | x | x | ≤4 | 8 | ≥16 | ≤8 | 16 | ≥32 |
| Cefepime^3^ | x | x | ≤2 | 4-8 (SDD) | ≥16 | ≤8 | - | ≥16 |
| Ceftazidime^3^ | x | x | ≤4 | 8 | ≥16 | 8 | - | 16 |
| Ceftazidime/Avibactam^3,6,7^ | x | x | ≤8/4 |  | ≥16/4 | ≤8/4 | - | 16/4 |
| Ceftolozane/Tazobactam | x | NA | NA | NA | NA | NA | NA | NA |
| Ceftriaxone^3^ | x | x | ≤1 | 2 | ≥4 | - | - | - |
| Ciprofloxacin^3,4^ | x | x | ≤ 0.25 | 0.5 | ≥ 1 | ≤0.5 | 1 | ≥ 2 |
| Ertapenem^3^ | x | x | ≤0.5 | 1 | ≥ 2 | - | - | - |
| ESBL confirmation^5^ | x | NA | NA | NA | NA | NA | NA | NA |
| Gentamicin^5^ | x | x | 4 | 8 | 16 | ≤4 | 8 | ≥16 |
| Imipenem | x | NA | NA | NA | NA | NA | NA | NA |
| Levofloxacin | x | NA | NA | NA | NA | NA | NA | NA |
| Meropenem^3,4^ | x | x | ≤1 | 2 | ≥4 | ≤2 | 4 | ≥8 |
| Meropenem/Vaborbactam | x | NA | NA | NA | NA | NA | NA | NA |
| Piperacillin/Tazobactam | x | x | ≤8/4 | 16/4 | ≥32/4 | ≤8/4 | 16/4 (SDD) | ≥32/4 |
| Tobramycin | x | x | ≤4 | 8 | ≥16 | ≤4 | 8 | ≥16 |
| Trimethoprim/Sulfamethoxazole^3,6^ | x | x | ≤2/38 | - | ≥4/76 | - | - | - |

^1^Antimicrobial susceptibility was interpreted using FDA STIC breakpoints published on June 20, 2024 where available otherwise CLSI M100 34th Ed (2024) was used.

^2^VITEK® REVEAL™ RUO panel was used in the study.

CLSI M100 34th Ed (2024) standard was used when recognized by the FDA STIC for the ^3^Enterobacterales group, and ^4^*Pseudomonas aeruginosa*.

^5^FDA cleared but not on the commercially available VITEK® REVEAL™ GN02 panel.

^6^RUO for Accelerate Pheno®.

^7^RUO for Accelerate Pheno® and not claimed for *Pseudomonas aeruginosa* therefore not analyzed.

NA (Not Applicable) – Six antimicrobial on the VITEK® REVEAL™ GN BC AST RUO panel but not included on the Accelerate Pheno® AST panel and excluded from evaluation; BPs (Breakpoints); RUO (Research Use Only); SDD (Susceptible dose-dependent); - means no breakpoints.

**TABLE S2** Samples collected during the study

| **Species** | **Number of Isolates** |
| --- | --- |
| **Evaluated Samples** | **128** |
| **Enterobacterales** | **115** |
| *E. coli* | 60 |
| *K. pneumoniae* group | 24 |
| *E. cloacae* complex | 10 |
| *P. mirabilis* | 8 |
| *S. marcescens* | 7 |
| *K. aerogenes* | 3 |
| *C. freundii* | 1 |
| *C. koseri* | 1 |
| *K. oxytoca* | 1 |
| ***P. aeruginosa*** | **13** |
| **Excluded samples** | **30** |
| **Total** | **158** |

A total of 158 de-identified, positive Gram-negative blood culture samples were enrolled in the study. 30 samples were excluded from analysis due to various exclusion criteria, with the remaining 128 analyzed samples consisting of 89.8% Enterobacterales species (*E. coli* (n=60), *K. pneumoniae* (n=24), *E. cloacae* complex (n=10), *Proteus* spp. (n=8), *S. marcescens* (n=7), *K. aerogenes* (n=3), *K. oxytoca* (n=1), and *Citrobacter* spp. (n=2)) and 10.2% *P. aeruginosa*.

**Table S3:** Performance of antimicrobials on the VITEK® REVEAL™ vs. Accelerate Pheno® systems for Enterobacterales by species^1^

|  | No. of strains with Accelerate Pheno® as reference | | | Agreement | | Discrepancies | | |
| --- | --- | --- | --- | --- | --- | --- | --- | --- |
|  | S | I | R | %EA (n/Total) | %CA (n/Total) | %VMD (n/R) | %MD (n/S) | %miD (n/Total) |
| ***E. coli* (n=60)** |  |  |  |  |  |  |  |  |
| Amikacin | 58 | 0 | 0 | 100.0 (58/58) | 100.0 (58/58) | - | 0.0 (0/58) | 0.0 (0/58) |
| Ampicillin/Sulbactam | 23 | 2 | 35 | 65.0 (60/60) | 55.0 (33/60) | 20.0 (7/35) | 0.0 (0/23) | 33.3 (20/60) |
| Aztreonam | 38 | 2 | 19 | 93.2 (55/59) | 89.8 (53/59) | 10.5 (2/19) | 5.3 (2/38) | 3.4 (2/59) |
| Cefepime | 43 | 2 | 15 | 96.7 (58/60) | 90.0 (54/60) | 0.0 (0/15) | 0.0 (0/43) | 10.0 (6/60) |
| Ceftazidime | 38 | 4 | 18 | 95.0 (57/60) | 88.3 (53/60) | 16.7 (3/18) | 0.0 (0/38) | 6.7 (4/60) |
| Ceftazidime/Avibactam | 60 | 0 | 0 | 100.0 (60/60) | 100.0 (60/60) | - | 0.0 (0/60) | 0.0 (0/60) |
| Ceftriaxone | 37 | 0 | 23 | 98.3 (59/60) | 98.3 (59/60) | 0.0 (0/23) | 2.7 (1/37) | 0.0 (0/60) |
| Ciprofloxacin | 37 | 1 | 22 | 98.3 (59/60) | 90.0 (54/60) | 0.0 (0/22) | 2.7 (1/37) | 8.3 (5/60) |
| Ertapenem | 60 | 0 | 0 | 100.0 (60/60) | 100.0 (60/60) | - | 0.0 (0/60) | 0.0 (0/60) |
| Gentamicin | 47 | 0 | 13 | 98.3 (59/60) | 98.3 (59/60) | 7.7 (1/13) | 0.0 (0/47) | 0.0 (0/60) |
| Meropenem | 59 | 0 | 0 | 100.0 (59/59) | 100.0 (59/59) | - | 0.0 (0/59) | 0.0 (0/59) |
| Piperacillin/Tazobactam | 53 | 0 | 4 | 93.0 (53/57) | 96.5 (55/57) | 25.0 (1/4) | 0.0 (0/53) | 1.8 (1/57) |
| Tobramycin | 45 | 1 | 10 | 100.0 (56/56) | 98.2 (55/56) | 0.0 (0/10) | 0.0 (0/45) | 1.8 (1/56) |
| Trimethoprim/Sulfamethoxazole | 31 | 0 | 0 | 100.0 (31/31) | 100.0 (31/31) | - | 0.0 (0/31) | 0.0 (0/31) |
| Total Antimicrobials | 629 | 12 | 159 | 95.4 (763/800) | 92.9 (743/800) | 8.8 (14/159) | 0.6 (4/629) | 4.9 (39/800) |
| ***K. pneumoniae* (n=24)** |  |  |  |  |  |  |  |  |
| Amikacin | 23 | 0 | 0 | 100.0 (23/23) | 100.0 (23/23) | - | 0.0 (0/23) | 0.0 (0/23) |
| Aztreonam | 18 | 0 | 6 | 100.0 (24/24) | 100.0 (24/24) | 0.0 (0/6) | 0.0 (0/18) | 0.0 (0/24) |
| Cefepime | 18 | 0 | 6 | 100.0 (24/24) | 100.0 (24/24) | 0.0 (0/6) | 0.0 (0/18) | 0.0 (0/24) |
| Ceftazidime | 18 | 1 | 4 | 100.0 (23/23) | 95.7 (22/23) | 0.0 (0/4) | 0.0 (0/18) | 4.3 (1/23) |
| Ceftazidime/Avibactam | 24 | 0 | 0 | 100.0 (24/24) | 100.0 (24/24) | - | 0.0 (0/24) | 0.0 (0/24) |
| Ceftriaxone | 18 | 0 | 5 | 100.0 (23/23) | 100.0 (23/23) | 0.0 (0/5) | 0.0 (0/18) | 0.0 (0/23) |
| Ciprofloxacin | 18 | 1 | 5 | 91.7 (22/24) | 91.7 (22/24) | 0.0 (0/5) | 0.0 (0/18) | 8.3 (2/24) |
| Ertapenem | 23 | 0 | 1 | 100.0 (24/24) | 95.8 (23/24) | 0.0 (0/1) | 0.0 (0/23) | 4.2 (1/24) |
| Gentamicin | 19 | 0 | 5 | 100.0 (24/24) | 95.8 (23/24) | 0.0 (0/5) | 0.0 (0/19) | 4.2 (1/24) |
| Meropenem | 22 | 0 | 0 | 90.9 (20/22) | 100.0 (22/22) | - | 0.0 (0/22) | 0.0 (0/22) |
| Piperacillin/Tazobactam | 17 | 0 | 0 | 100.0 (17/17) | 100.0 (17/17) | - | 0.0 (0/17) | 0.0 (0/17) |
| Tobramycin | 19 | 2 | 3 | 100.0 (24/24) | 95.8 (23/24) | 0.0 (0/3) | 0.0 (0/19) | 4.2 (1/24) |
| Trimethoprim/Sulfamethoxazole | 15 | 0 | 9 | 95.8 (23/24) | 100.0 (24/24) | 0.0 (0/9) | 0.0 (0/15) | 0.0 (0/24) |
| Total Antimicrobials | 252 | 4 | 44 | 98.3 (295/300) | 98.0 (294/300) | 0.0 (0/44) | 0.0 (0/252) | 2.0 (6/300) |
| ***E. cloacae* complex (n=10)** |  |  |  |  |  |  |  |  |
| Amikacin | 10 | 0 | 0 | 100.0 (10/10) | 100.0 (10/10) | - | 0.0 (0/10) | 0.0 (0/10) |
| Aztreonam | 7 | 0 | 3 | 100.0 (10/10) | 100.0 (10/10) | 0.0 (0/3) | 0.0 (0/7) | 0.0 (0/10) |
| Cefepime | 9 | 1 | 0 | 90.0 (9/10) | 90.0 (9/10) | - | 0.0 (0/9) | 10.0 (1/10) |
| Ceftazidime | 7 | 0 | 3 | 100.0 (10/10) | 100.0 (10/10) | 0.0 (0/3) | 0.0 (0/7) | 0.0 (0/10) |
| Ceftazidime/Avibactam | 9 | 0 | 0 | 100.0 (9/9) | 100.0 (9/9) | - | 0.0 (0/9) | 0.0 (0/9) |
| Ceftriaxone | 7 | 0 | 3 | 100.0 (10/10) | 100.0 (10/10) | 0.0 (0/3) | 0.0 (0/7) | 0.0 (0/10) |
| Meropenem | 7 | 0 | 0 | 100.0 (7/7) | 100.0 (7/7) | - | 0.0 (0/7) | 0.0 (0/7) |
| Tobramycin | 10 | 0 | 0 | 100.0 (10/10) | 100.0 (10/10) | - | 0.0 (0/10) | 0.0 (0/10) |
| Total Antimicrobials | 66 | 1 | 9 | 98.7 (75/76) | 98.7 (75/76) | 0.0 (0/9) | 0.0 (0/66) | 1.3 (1/76) |
| **Other Enterobacterales (n=21)** |  |  |  |  |  |  |  |  |
| Amikacin | 18 | 0 | 0 | 94.4 (17/18) | 100.0 (18/18) | - | 0.0 (0/18) | 0.0 (0/18) |
| Ampicillin/Sulbactam | 5 | 3 | 1 | 55.6 (5/9) | 44.4 (4/9) | 100.0 (1/1) | 0.0 (0/5) | 44.4 (4/9) |
| Aztreonam | 2 | 0 | 0 | 100.0 (2/2) | 100.0 (2/2) | - | 0.0 (0/2) | 0.0 (0/2) |
| Cefepime | 5 | 0 | 0 | 100.0 (5/5) | 100.0 (5/5) | - | 0.0 (0/5) | 0.0 (0/5) |
| Ceftazidime | 2 | 2 | 1 | 100.0 (5/5) | 60.0 (3/5) | 0.0 (0/1) | 0.0 (0/2) | 40.0 (2/5) |
| Ceftazidime/Avibactam | 13 | 0 | 0 | 100.0 (13/13) | 100.0 (13/13) | - | 0.0 (0/13) | 0.0 (0/13) |
| Ceftriaxone | 10 | 0 | 2 | 100.0 (12/12) | 100.0 (12/12) | 0.0 (0/2) | 0.0 (0/10) | 0.0 (0/12) |
| Ciprofloxacin | 10 | 1 | 6 | 94.1 (16/17) | 88.2 (15/17) | 0.0 (0/6) | 10.0 (1/10) | 5.9 (1/17) |
| Ertapenem | 8 | 0 | 0 | 100.0 (8/8) | 100.0 (8/8) | - | 0.0 (0/8) | 0.0 (0/8) |
| Gentamicin | 19 | 1 | 1 | 95.2 (20/21) | 95.2 (20/21) | 100.0 (1/1) | 0.0 (0/19) | 0.0 (0/21) |
| Meropenem | 13 | 0 | 0 | 100.0 (13/13) | 100.0 (13/13) | - | 0.0 (0/13) | 0.0 (0/13) |
| Piperacillin/Tazobactam | 1 | 0 | 0 | 0.0 (0/1) | 100.0 (1/1) | - | 0.0 (0/1) | 0.0 (0/1) |
| Tobramycin | 21 | 0 | 0 | 95.2 (20/21) | 100.0 (21/21) | - | 0.0 (0/21) | 0.0 (0/21) |
| Trimethoprim/Sulfamethoxazole | 3 | 0 | 0 | 100.0 (3/3) | 100.0 (3/3) | - | 0.0 (0/3) | 0.0 (0/3) |
| Total Antimicrobials | 130 | 7 | 11 | 93.9 (139/148) | 93.2 (138/148) | 18.2 (2/11) | 0.8 (1/130) | 4.7 (7/148) |

^1^Results shown are prior to discrepancy resolution. Antimicrobial performance on the VITEK® REVEAL™ vs. Accelerate Pheno® systems are grouped by the following species: *E. coli* (n=60), *K. pneumonia* (n=24), *E. cloacae* complex (n=10), and Other Enterobacterales (group includes *C. koseri* (n=1), *C. freundii* (n=1), *K. aerogenes* (n=3), *K. oxytoca* (n=1), *P. mirabilis* (n=8), and *S. marcescens* (n=7))

**TABLE S4** Detailed MIC and interpretations for VITEK® REVEAL™, Accelerate Pheno®, and BMD for Strains with discrepancies

| **Sample ID** | **Species** | **Antimicrobial** | **VITEK® REVEAL™  MIC** | **VITEK® REVEAL™ Interpretation** | **Accelerate Pheno®  MIC** | **Accelerate Pheno® Interpretation** | **BMD MIC (Mode)** | **BMD Interpretation** | **CA  (VITEK® REVEAL™ vs. BMD)** | **CA (Accelerate Pheno® vs. BMD)** | **No CA with BMD** |
| --- | --- | --- | --- | --- | --- | --- | --- | --- | --- | --- | --- |
| **Very Major Discrepancies** | | | | | | | | | | | |
| 03-020 | *E. coli* | Ampicillin/Sulbactam | 8 | S | 32 | R |  |  | 0 | 0 | 1 |
| 03-023 | *E. coli* | Ampicillin/Sulbactam | 8 | S | >=64 | R | 32 | R | 0 | 1 | 0 |
| 03-027 | *E. coli* | Ampicillin/Sulbactam | 8 | S | >=64 | R | 32 | R | 0 | 1 | 0 |
| 03-053 | *E. coli* | Ampicillin/Sulbactam | 8 | S | >=64 | R | 16 | I | 0 | 0 | 1 |
| 03-116 | *K. oxytoca* | Ampicillin/Sulbactam | 8 | S | 32 | R | 8 | S | 1 | 0 | 0 |
| 03-119 | *E. coli* | Ampicillin/Sulbactam | 8 | S | 32 | R | 8 | S | 1 | 0 | 0 |
| 03-147 | *E. coli* | Ampicillin/Sulbactam | 8 | S | 32 | R | 8 | S | 1 | 0 | 0 |
| 03-153 | *E. coli* | Ampicillin/Sulbactam | 8 | S | >=64 | R | 16 | I | 0 | 0 | 1 |
| 03-109 | *E. coli* | Aztreonam | <=4 | S | 16 | R | 8 | I | 0 | 0 | 1 |
| 03-127 | *E. coli* | Aztreonam | <=4 | S | 16 | R | 4 | S | 1 | 0 | 0 |
| 03-136 | *P. aeruginosa* | Aztreonam | 8 | S | >=64 | R | 8 | S | 1 | 0 | 0 |
| 03-072 | *E. coli* | Ceftazidime | <=4 | S | >=32 | R | 4 | S | 1 | 0 | 0 |
| 03-109 | *E. coli* | Ceftazidime | <=4 | S | 16 | R | 4 | S | 1 | 0 | 0 |
| 03-149 | *E. coli* | Ceftazidime | <=4 | S | >=32 | R | 8 | I | 0 | 0 | 1 |
| 03-075 | *P. mirabilis* | Gentamicin | 4 | S | 16 | R | 8 | I | 0 | 0 | 1 |
| 03-095 | *E. coli* | Gentamicin | <=2 | S | 16 | R | 0.5 | S | 1 | 0 | 0 |
| 03-023 | *E. coli* | Piperacillin/Tazobactam | <=2 | S | 32 | R | 16 | I | 0 | 0 | 1 |
| **Overall (n)** |  |  |  |  |  |  |  |  | **8** | **2** | **7** |
| **Overall (%)** |  |  |  |  |  |  |  |  | **47.1** | **11.8** | **41.2** |
| **Major Discrepancies** | | | | | | | | | | | |
| 03-095 | *E. coli* | Aztreonam | 16 | R | 2 | S | 0.25 | S | 0 | 1 | 0 |
| 03-097 | *E. coli* | Aztreonam | 16 | R | 4 | S | 8 | I | 0 | 0 | 1 |
| 03-095 | *E. coli* | Ceftriaxone | >4 | R | 0.5 | S | 0.06 | S | 0 | 1 | 0 |
| 03-149 | *E. coli* | Ciprofloxacin | 1 | R | <=0.25 | S | 0.25 | S | 0 | 1 | 0 |
| 03-074 | *P. aeruginosa* | Cefepime | 16 | R | 8 | S | 16 | R | 1 | 0 | 0 |
| 03-036 | *S. marcescens* | Ciprofloxacin | >2 | R | <=0.25 | S | 0.015 | S | 0 | 1 | 0 |
| **Overall (n)** |  |  |  |  |  |  |  |  | **1** | **4** | **1** |
| **Overall (%)** |  |  |  |  |  |  |  |  | **16.7** | **66.7** | **16.7** |
| **Minor Discrepancies** | | | | | | | | | | | |
| 03-003 | *P. mirabilis* | Ampicillin/Sulbactam | 16 | I | <=4 | S | 16 | I | 1 | 0 | 0 |
| 03-012 | *E. coli* | Ampicillin/Sulbactam | 16 | I | >=64 | R | 32 | R | 0 | 1 | 0 |
| 03-014 | *E. coli* | Ampicillin/Sulbactam | 8 | S | 16 | I | 8 | S | 1 | 0 | 0 |
| 03-024 | *E. coli* | Ampicillin/Sulbactam | 16 | I | >=64 | R | 32 | R | 0 | 1 | 0 |
| 03-028 | *E. coli* | Ampicillin/Sulbactam | 16 | I | >=64 | R | 32 | R | 0 | 1 | 0 |
| 03-031 | *E. coli* | Ampicillin/Sulbactam | 16 | I | 32 | R | 16 | I | 1 | 0 | 0 |
| 03-041 | *P. mirabilis* | Ampicillin/Sulbactam | <=4 | S | 16 | I | 1 | S | 1 | 0 | 0 |
| 03-042 | *E. coli* | Ampicillin/Sulbactam | 16 | I | >=64 | R | 32 | R | 0 | 1 | 0 |
| 03-043 | *E. coli* | Ampicillin/Sulbactam | 16 | I | >=64 | R | 16 | I | 1 | 0 | 0 |
| 03-046 | *E. coli* | Ampicillin/Sulbactam | 16 | I | 32 | R | 32 | R | 0 | 1 | 0 |
| 03-049 | *E. coli* | Ampicillin/Sulbactam | 16 | I | >=64 | R | 32 | R | 0 | 1 | 0 |
| 03-061 | *E. coli* | Ampicillin/Sulbactam | 16 | I | >=64 | R | 16 | I | 1 | 0 | 0 |
| 03-063 | *E. coli* | Ampicillin/Sulbactam | 16 | I | >=64 | R | 32 | R | 0 | 1 | 0 |
| 03-064 | *E. coli* | Ampicillin/Sulbactam | 16 | I | >=64 | R | 8 | S | 0 | 0 | 1 |
| 03-075 | *P. mirabilis* | Ampicillin/Sulbactam | <=4 | S | 16 | I | 4 | S | 1 | 0 | 0 |
| 03-079 | *E. coli* | Ampicillin/Sulbactam | 16 | I | 32 | R | 32 | R | 0 | 1 | 0 |
| 03-092 | *E. coli* | Ampicillin/Sulbactam | 16 | I | >=64 | R | 16 | I | 1 | 0 | 0 |
| 03-095 | *E. coli* | Ampicillin/Sulbactam | 16 | I | 32 | R | 16 | I | 1 | 0 | 0 |
| 03-096 | *E. coli* | Ampicillin/Sulbactam | 8 | S | 16 | I | 8 | S | 1 | 0 | 0 |
| 03-100 | *P. mirabilis* | Ampicillin/Sulbactam | >16 | R | 16 | I | 16 | I | 0 | 1 | 0 |
| 03-102 | *E. coli* | Ampicillin/Sulbactam | 16 | I | >=64 | R | 16 | I | 1 | 0 | 0 |
| 03-135 | *E. coli* | Ampicillin/Sulbactam | 16 | I | >=64 | R | 32 | R | 0 | 1 | 0 |
| 03-138 | *E. coli* | Ampicillin/Sulbactam | 16 | I | >=64 | R | 32 | R | 0 | 1 | 0 |
| 03-149 | *E. coli* | Ampicillin/Sulbactam | 16 | I | >=64 | R | 16 | I | 1 | 0 | 0 |
| 03-025 | *E. coli* | Aztreonam | <=4 | S | 8 | I | 8 | I | 0 | 1 | 0 |
| 03-030 | *P. aeruginosa* | Aztreonam | 8 | S | 16 | I | 8 | S | 1 | 0 | 0 |
| 03-074 | *P. aeruginosa* | Aztreonam | 16 | I | >=64 | R | 32 | R | 0 | 1 | 0 |
| 03-079 | *E. coli* | Aztreonam | 16 | R | 8 | I | 32 | R | 1 | 0 | 0 |
| 03-015 | *E. cloacae complex* | Cefepime | <=2 | S | 8 | I | 2 | S | 1 | 0 | 0 |
| 03-025 | *E. coli* | Cefepime | 4 | I | <=1 | S | 4 | I | 1 | 0 | 0 |
| 03-072 | *E. coli* | Cefepime | 16 | R | 8 | I | 2 | S | 0 | 0 | 1 |
| 03-097 | *E. coli* | Cefepime | 8 | I | <=1 | S | 4 | I | 1 | 0 | 0 |
| 03-109 | *E. coli* | Cefepime | 4 | I | 2 | S | 8 | I | 1 | 0 | 0 |
| 03-127 | *E. coli* | Cefepime | 4 | I | 2 | S | 4 | I | 1 | 0 | 0 |
| 03-149 | *E. coli* | Cefepime | 8 | I | 16 | R | 32 | R | 0 | 1 | 0 |
| 03-013 | *K. aerogenes* | Ceftazidime | <=4 | S | 8 | I | 2 | S | 1 | 0 | 0 |
| 03-025 | *E. coli* | Ceftazidime | <=4 | S | 8 | I | 8 | I | 0 | 1 | 0 |
| 03-053 | *E. coli* | Ceftazidime | <=4 | S | 8 | I | 0.12 | S | 1 | 0 | 0 |
| 03-079 | *E. coli* | Ceftazidime | <=4 | S | 8 | I | 8 | I | 0 | 1 | 0 |
| 03-082 | *K. pneumoniae* | Ceftazidime | 16 | R | 8 | I | 32 | R | 1 | 0 | 0 |
| 03-127 | *E. coli* | Ceftazidime | <=4 | S | 8 | I | 4 | S | 1 | 0 | 0 |
| 03-158 | *K. aerogenes* | Ceftazidime | <=4 | S | 8 | I | 1 | S | 1 | 0 | 0 |
| 03-007 | *K. pneumoniae* | Ciprofloxacin | 0.5 | I | <=0.25 | S | 0.5 | I | 1 | 0 | 0 |
| 03-010 | *K. pneumoniae* | Ciprofloxacin | <=0.25 | I | <=0.5 | S | 0.5 | I | 1 | 0 | 0 |
| 03-024 | *E. coli* | Ciprofloxacin | <=0.25 | S | <=0.5 | I | 0.008 | S | 1 | 0 | 0 |
| 03-032 | *P. mirabilis* | Ciprofloxacin | <=0.25 | S | 0.5 | I | 0.03 | S | 1 | 0 | 0 |
| 03-079 | *E. coli* | Ciprofloxacin | 0.5 | I | <=0.25 | S | 0.5 | I | 1 | 0 | 0 |
| 03-087 | *E. coli* | Ciprofloxacin | <=0.25 | I | <=0.5 | S | 0.12 | S | 0 | 1 | 0 |
| 03-119 | *E. coli* | Ciprofloxacin | <=0.25 | I | <=0.5 | S | 0.25 | S | 0 | 1 | 0 |
| 03-134 | *E. coli* | Ciprofloxacin | <=0.25 | I | <=0.5 | S | 0.25 | S | 0 | 1 | 0 |
| 03-007 | *K. pneumoniae* | Ertapenem | 8 | I | 16 | R | 8 | R | 0 | 1 | 0 |
| 03-139 | *K. pneumoniae* | Gentamicin | <=2 | I | 8 | R | 64 | R | 0 | 1 | 0 |
| 03-150 | *P. aeruginosa* | Gentamicin | 4 | S | 8 | I | 4 | S | 1 | 0 | 0 |
| 03-131 | *P. aeruginosa* | Meropenem | 2 | I | 4 | R | 4 | I | 1 | 0 | 0 |
| 03-136 | *P. aeruginosa* | Meropenem | 16 | S | 128 | I | 2 | S | 1 | 0 | 0 |
| 03-039 | *E. coli* | Piperacillin/Tazobactam | >8 | I | 8 | R | 16 | I | 1 | 0 | 0 |
| 03-082 | *K. pneumoniae* | Tobramycin | 8 | R | 16 | I | 16 | R | 1 | 0 | 0 |
| 03-106 | *E. coli* | Tobramycin | 8 | I | 16 | R | 8 | I | 1 | 0 | 0 |
| **Overall (n)** |  |  |  |  |  |  |  |  | **35** | **21** | **2** |
| **Overall (%)** |  |  |  |  |  |  |  |  | **60.3** | **36.2** | **3.4** |
